# Unexpected dietary preferences of Eurasian Spoonbills in the Dutch Wadden Sea: spoonbills mainly feed on small fish not shrimp

**DOI:** 10.1007/s10336-018-1551-2

**Published:** 2018-03-24

**Authors:** Jeltje Jouta, Petra de Goeij, Tamar Lok, Estefania Velilla, Cornelis J. Camphuysen, Mardik Leopold, Henk W. van der Veer, Han Olff, Otto Overdijk, Theunis Piersma

**Affiliations:** 1 Conservation Ecology Group, Groningen Institute for Evolutionary Life Sciences (GELIFES), University of Groningen, P.O. Box 11103, 9700 CC Groningen, The Netherlands; 2 Department of Coastal Systems, NIOZ Royal Netherlands Institute for Sea Research and Utrecht University, P.O. Box 59, 1790 AB Den Burg, Texel The Netherlands; 3 Werkgroep Lepelaar, Visserspad 10, 9142 VN Moddergat, The Netherlands; 4 Biostatistics and Population Biology, Centre d’Ecologie Fonctionnelle et Evolutive, UMR 5175, Montpellier, France; 5 Department of Ecological Science, Faculty of Earth and Life Sciences, VU University Amsterdam, De Boelelaan 1085, 1081 HV Amsterdam, The Netherlands; 6 Present Address: Ecosystems, Wageningen IMARES, P.O. Box 57, 1780 AB Den Helder, The Netherlands

**Keywords:** *Platalea leucorodia leucorodia*, Regurgitate analysis, Restoration, Stable isotope analysis in R, Intertidal, Bayesian mixing models

## Abstract

After an historical absence, over the last decades Eurasian Spoonbills *Platalea leucorodia leucorodia* have returned to breed on the barrier islands of the Wadden Sea. The area offers an abundance of predator-free nesting habitat, low degrees of disturbance, and an extensive intertidal feeding area with increasing stocks of brown shrimp *Crangon crangon*, the assumed main prey of *P. leucorodia leucorodia*. Nevertheless, newly established and expanding colonies of spoonbills have surprisingly quickly reached plateau levels. Here we tested the often stated assertion that spoonbills mainly rely on brown shrimp as food, by quantifying the diet of chicks on the basis of regurgitates and by analysis of blood isotopes using stable isotope Bayesian mixing models. Both methods showed that, rather than brown shrimp being the staple food of spoonbill chicks, small flatfish (especially plaice *Pleuronectes platessa*) and gobies (*Pomatoschistus* spp.) were their main prey. Unlike shrimp, small flatfish have been reported to be rather scarce in the Wadden Sea in recent years, which may explain the rapid saturation of colony size due to food-related density-dependent recruitment declines of growing colonies. By way of their diet and colony growth characteristics, spoonbills may thus indicate the availability of small fish in the Wadden Sea. We predict that the recovery to former densities of young flatfish and other juvenile/small fish in the Wadden Sea will be tracked by changing diets (more fish) and an increase in the size of Eurasian Spoonbill colonies across the Wadden Sea.

## Zusammenfassung

**Unerwartete Nahrungsvorlieben bei Löfflern** ***Platalea leucorodia leucorodia*** **im niederländischen Wattenmeer: Statt Garnelen fressen Löffler hauptsächlich kleine Fische**

Nach längerer Abwesenheit in der Vergangenheit ist der Löffler *Platalea leucorodia leucorodia* während der letzten Jahrzehnte als Brutvogel auf die vorgelagerten Inseln des Wattenmeeres zurückgekehrt. Dieser Lebensraum bietet reichlich prädatorfreies Nisthabitat, ein geringes Maß an Störungen sowie räumlich scheinbar grenzenlose Nahrungsflächen in der Gezeitenzone mit zunehmenden Beständen an Nordseegarnelen *Crangon crangon*, welche als die Hauptbeute der Löffler gelten. Dennoch haben die neugegründeten und sich ausdehnenden Kolonien überraschend schnell ihre Sättigungsgrenze erreicht. Hier überprüften wir die oft geäußerte Behauptung, dass Löffler hauptsächlich auf Nordseegarnelen als Nahrung angewiesen sind, indem wir die Kükennahrung anhand von Speiballen und durch Analysen der Isotope im Blut mittels Bayes’scher gemischter Modelle stabiler Isotope quantifizierten. Bei beiden Methoden zeigte sich, dass nicht, wie erwartet, vorwiegend Nordseegarnelen an die Löfflerküken verfüttert wurden, sondern dass kleine Plattfische (besonders Scholle *Pleuronectes platessa*) und Grundeln (*Pomatoschistus* spp.) die Hauptbeute darstellten. Anders als die Garnelen sind kleine Plattfische im Wattenmeer in den letzten Jahren selten geworden, was aufgrund nahrungsbedingter und dichteabhängiger Abnahmen der Rekrutierungsraten wachsender Kolonien das schnelle Erreichen der Sättigungsgrenze bezüglich der Koloniegröße erklären könnte. Aufgrund ihrer Ernährungsweise und ihrer Koloniewachstumseigenschaften könnten Löffler so die Verfügbarkeit kleiner Fische im Wattenmeer anzeigen. Wir sagen voraus, dass eine Bestandserholung junger Plattfische und anderer juveniler beziehungsweise kleiner Fische zu früheren Dichten im Wattenmeer an einer veränderten Nahrungszusammensetzung (mehr Fische) und einer wachsenden Koloniegröße der Löffler im gesamten Wattenmeer erkennbar sein wird.

## Introduction

The Wadden Sea, the area of shallows and intertidal flats between the northwestern coast of the European mainland and the barrier islands which create a border to the North Sea, provides a vast habitat for marine and estuarine species, including those that connect the Wadden Sea with ecosystems elsewhere on the globe, i.e. migratory shorebirds (Swennen 1976; van de Kam et al. 2004; Reise et al. 2010; van Roomen et al. 2012). This ecosystem is subject to many external forces, many of the human ones contributing to the degradation of ecosystem functioning (de Jonge and Essink 1993; Wolff 2005; Eriksson et al. 2010). In recent decades, following recognition of the Wadden Sea's importance, such as its RAMSAR status and, more recently, its designation as a UNESCO World Heritage Site, attempts have been made to conserve and restore the biodiversity and ecosystem functioning of this sea (Boere and Piersma 2012).

The return of the Eurasian Spoonbill *Platalea leucorodia leucorodia* as a breeding bird to the Wadden Sea barrier islands, after an historical absence of many centuries due to human persecution (de Goeij et al. 2015), counts as a tangible result of successful conservation measures. From the late 1960s onwards, the number of spoonbill breeding pairs increased exponentially in the Dutch Wadden Sea (de Goeij et al. 1985; Lok et al. 2009; Oudman et al. 2017), as a result of favourable circumstances on the Wadden Sea islands due to the enforced protection of foraging and breeding areas (de Goeij et al. 1985; Kemper 1986a; van der Hut 1992). This increase in numbers has been partly due to resettlement from mainland colonies threatened by red fox predation, from immigration due to other factors, and also a result of local recruitment (Lok et al. 2009). Indeed, the Wadden Sea seems to provide everything that reproductively active spoonbills need: plenty of suitable nesting places with little or no predation, very low degrees of disturbance, and extensive foraging areas in the form of shallow gullies and tidal flats.

It has therefore been surprising that newly established and expanding colonies in the Wadden Sea have quickly reached plateau levels (Lok et al. 2009; Oudman et al. 2017), the increase of the total breeding numbers being driven to a large extent by the formation of new colonies near previously unoccupied areas of intertidal flats. Growing colonies show signs of density dependence as (1) the number of fledglings per nest has declined with colony size (Lok et al. 2009; Oudman et al. 2017), and (2) the post-fledging survival rates of spoonbills have declined with an increase of overall population size (Lok et al. 2013). In view of the large unused areas of what appears to be high-quality breeding habitat, it has been suggested that food might be the factor causing density dependence and limiting population size in the Eurasian Spoonbill (Oudman et al. 2017).

**Fig. 1:**
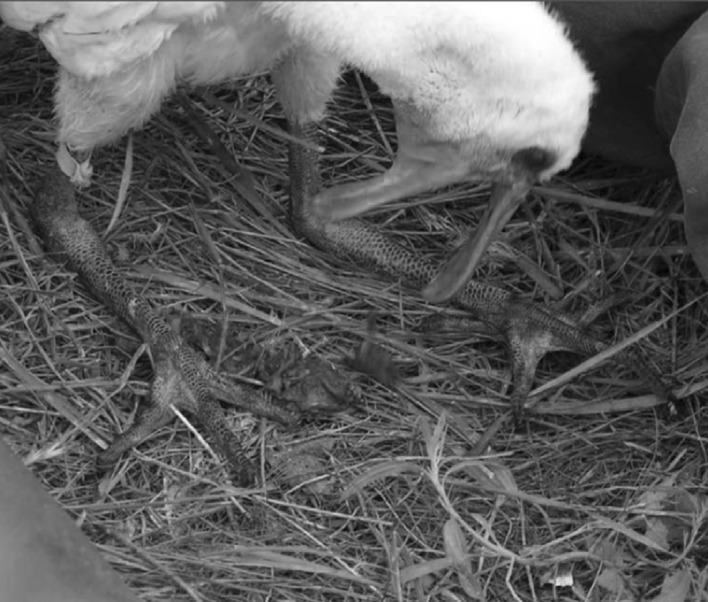
A spoonbill chick producing a regurgitate upon being held after capture. At first sight such regurgitates only show the remains of shrimp

Shrimp *Crangon crangon* have been repeatedly reported as being the main prey of spoonbills, especially during the chick-rearing period (Tinbergen 1933; Kemper 1986a, b; Wintermans and Wymenga 1996; Altenburg and Wymenga, 1997; Fig. 1). However, de Goeij et al. (1985) indicated that a high availability of young plaice in pools in the Wadden Sea during low tide would provide easy prey for spoonbills. The fact that the food of spoonbills might be limiting, and that several colonies reached plateau levels more than 10 years ago (e.g. on the islands of Terschelling and Schiermonnikoog), can possibly be explained either by (1) shrimp becoming more abundant (Tulp et al. 2012), but shrimp not actually being the staple food of these birds; or (2) by shrimp availability actually being lower than thought, e.g. as a result of high fishing pressure, as reported by Tulp et al. (2016). In this study we aim to examine these possibilities by studying the diet of nestling spoonbills across the colonies of the Dutch Wadden Sea, using both regurgitates and isotopic Bayesian mixing models (stable isotope analysis in R; SIAR) based on stable carbon (δ^13^C) and nitrogen (δ^15^N) isotopes in bird blood and prey tissue samples to estimate the diet of chicks. SIAR was used to model the isotopic (food web) position of spoonbills relative to their prey by using δ^13^C and δ^15^N; δ^13^C is useful for discrimination between marine and terrestrial organisms, while δ^15^N is useful when studying trophic position (Hobson and Welch 1992; Polis and Hurd 1995; Post 2002). By identifying the staple foods of spoonbills, we can suggest the best conservation management practices for them in the Wadden Sea.

## Materials and methods

Spoonbills are tactile foragers and have altricial chicks that are fed by the parents (Hancock et al. 2010). After a breeding period of about 25 days, during which both parents incubate the eggs, most chicks hatch from early to mid-May (Lok et al. 2017). During the breeding season spoonbills are bound to the nest and therefore restricted to a foraging range of less than 30–40 km from the breeding colony (Altenburg and Wymenga 1997). Although this foraging range allows them to forage in both marine and freshwater resources, chicks appear mainly to be fed with marine prey likely captured in the Wadden Sea (El-Hacen et al. 2014).

The diet of spoonbill chicks in colonies on the barrier islands of the Wadden Sea was assessed from regurgitates and from blood stable isotope analyses. Whereas regurgitates reflect the diet on the day of collection, stable isotope analyses indicate the diet integrated over longer periods: a few days if based on blood plasma, a few weeks if based on red blood cells (RBC) (e.g. Dietz et al. 2010; Hahn et al. 2012). Both methods are ideal for diet reconstruction, with the caveat that small prey and easily digestible prey may be missed when using diet reconstruction based on regurgitates, while the isotopic mixing model SIAR is an indirect method and requires precise assumptions (e.g. about discrimination factors and selection of potential prey for SIAR).

**Fig. 2:**
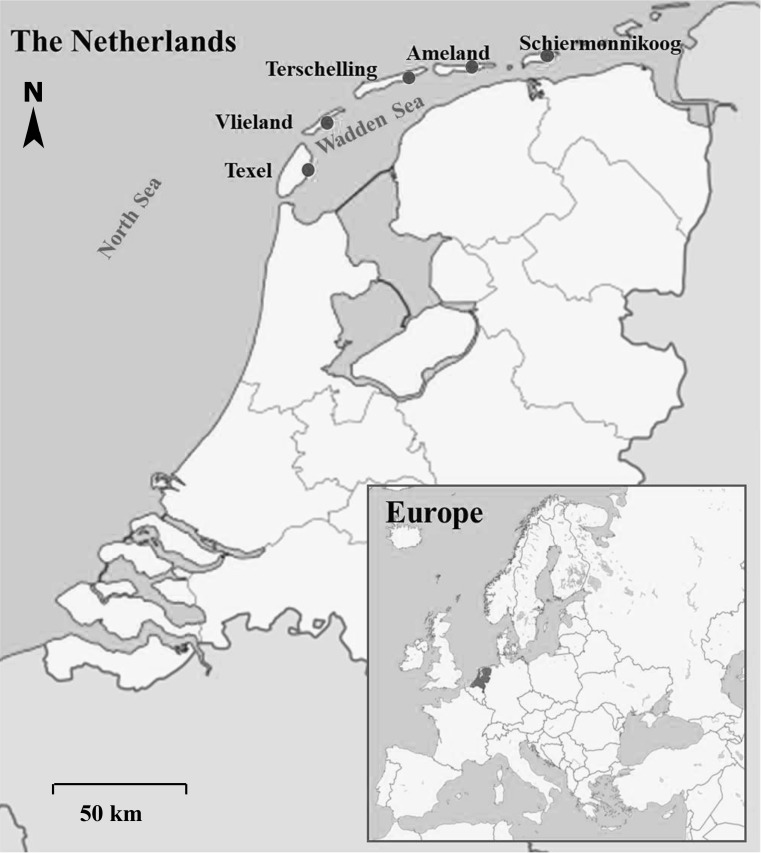
Map with overview of the Eurasian Spoonbill colonies on the five Dutch Wadden Sea islands where spoonbill samples were collected

From 17 May to 12 July 2012 and 12 June–7 August 2013, a total of 301 chicks aged 15–35 days were examined in colonies on five islands in the Dutch Wadden Sea (Fig. 2). Within 1 h after capture the birds were colour-ringed, body size measures were taken (see Lok et al. 2014) and a blood sample of 150–400 µl was taken from the brachial vein in heparinized capillaries. Within 3 h after sampling, blood plasma and RBC were separated in Eppendorf cups in a haematocrit centrifuge (Sigma 1–13 microfuge; 6 min at 5000 r.p.m.). Plasma and RBC were pipetted into separate glass vials. Samples were transported in a bag with cooling elements for maximally 4 h before storage at − 20 °C until analysis. To obtain the isotope values of potential prey, 209 food items were collected from 12 April to 17 May 2012 and 2 May–12 July 2013 in all potential feeding habitats of spoonbills. Prey collection occurred at locations where spoonbills were foraging at that moment or were known to forage frequently. The proportions of prey species as calculated by regurgitate analyses was used as a prior for prey selected in the stable isotopic based diet reconstruction SIAR (all prey species that compromised > 2% of the total diet). In order to limit the number of prey input into SIAR, we combined all important prey species in freshwater and mixture sources, while using all important prey of the marine water source. Prey categorized under ‘mixture’ are prey that occur in marine, brackish and freshwater habitat types. An overview of the prey species used for diet reconstruction with the help of isotopic mixing models (SIAR) is given in Table 1. C isotopes of prey were normalized a posteriori for the effect of lipid concentration, using a correction based on the C:N ratio given by Post et al. (2007) (Table 1).

**Table 1:** Mean stable isotope values of nitrogen (*δ*^15^*N*) and carbon (*δ*^13^*C*) of prey used as input for diet reconstruction in the stable isotope mixing model (stable isotope analysis in R; SIAR)

Water type	Island	Species	δ^15^N		δ^13^C		N	C:N		δ^13^C_lipid-corr_	
			Mean	SE	Mean	SE	N	Ratio	SE	Mean	SE
Marine	Texel	*Crangon crangon*	13.2	0.4	− 14.9	0.6	20	3.6	0.06	− 14.6	0.6
		*Pleuronectes platessa*	14.0	0.2	− 16.2	0.5	19	3.4	0.04	− 16.1	0.5
		*Pomatoschistus microps*	15.2	0.5	− 14.3	0.2	2	3.6	0.08	− 14.0	0.3
	Vlieland	*Crangon crangon*	12.2	0.2	− 13.2	0.2	7	3.7	0.06	− 12.8	0.2
		*Pleuronectes platessa*	11.6	0.3	− 14.5	0.3	4	3.5	0.06	− 14.4	0.3
		*Pomatoschistus microps*	14.1	0.6	− 15.4	0.8	3	4.0	0.06	15.1	1.3
	Terschelling	*Crangon crangon*	12.9	0.2	− 14.4	0.1	17	3.7	0.03	− 14.1	0.1
		*Pleuronectes platessa*	12.3	0.2	− 15.8	0.2	8	3.7	0.03	− 15.5	0.2
		*Pomatoschistus microps*	14.9	0.2	− 15.8	0.5	7	4.1	0.08	− 15.0	0.5
	Ameland^a^	*Crangon crangon*	12.8	0.2	− 14.6	0.3	56	3.7	0.02	− 14.3	0.3
		*Pleuronectes platessa*	13.0	0.2	− 16.4	0.4	38	3.5	0.03	− 16.2	0.4
		*Pomatoschistus microps*	14.7	0.2	− 15.5	0.4	12	4.0	0.07	− 14.8	0.3
	Schiermonnikoog	*Crangon crangon*	12.3	0.2	− 15.1	0.8	11	3.7	0.02	− 14.8	0.8
		*Pleuronectes platessa*	11.8	0.4	− 19.7	1.4	5	3.7	0.06	− 19.3	1.4
		*Pomatoschistus microps*	14.7	0.2	− 15.5	0.4	12	4.0	0.07	− 14.8	0.3
Mixture	All islands	Total^b^	12.8	0.5	− 24.2	0.8	44	4.3	0.2	− 23.2	0.8
		*Gasterosteus aculeatus* (90.8%)	12.4	0.5	− 24.9	0.8	40	4.4	2.2	− 23.8	0.9
		*Osmerus eperlanus* (9.2%)	16.3	0.3	− 17.1	0.2	4	3.2	0.02	− 17.2	0.3
Freshwater	All islands	Total^b^	15.9	0.8	− 27.5	0.4	46	3.4	0.06	− 27.5	0.3
		*Perca fluviatilis* (24.1%)	18.0	0.2	− 26.7	0.2	7	3.2	0.01	− 26.9	0.2
		*Pungitius pungitius* (19.4%)	7.8	0.4	− 30.2	0.5	24	3.9	0.07	− 29.7	0.4
		*Rutilus rutilus* (56.5%)	17.8	0.3	− 26.8	0.4	15	3.2	0.02	− 26.9	0.4

Selected prey species contributed > 2% of the diet assessed with regurgitate analyses. C isotopes were normalized for the effect of lipid concentration (*δ*^13^*C*_*lipid-corr*_), using a correction based on the C:N ratio given by Post et al. (2007)

^a^Because low numbers of main prey species were collected on Ameland, mean values of all islands were used

^b^Dietary ratios of the main prey based on the regurgitate analysis (> 2%; see Table 2) were used to calculate the mean stable isotope values of Mixture and Freshwater for prey input in SIAR

Stable isotope values of spoonbill chicks are shown in Table 2. As explained by Cherel et al. (2005), lipid extraction of plasma is required to measure adequate δ^13^C plasma values, especially since the C:N_plasma_ ratios of spoonbill chicks in this study are high (> 4.0). Although lipid correction is needed, we were not able to repeat the analyses with lipid-extracted samples. We did not find an a posteriori lipid correction model to ‘normalize’ bird plasma for the lipid contribution.

**Table 2:** Mean stable isotope values of spoonbill chicks used as an input for diet reconstruction via SIAR

Island	Tissue	δ^15^N		δ^13^C		TOC		TN		C:N	
		Mean	SE	Mean	SE	Mean	SE	Mean	SE	Mean	SE
Texel (*n* = 48)	Cells	14.82	0.14	− 16.31	0.23	47.86	0.19	14.96	0.07	3.20	0.01
	Plasma	16.41	0.15	− 16.53	0.20	42.66	0.27	9.99	0.10	4.28	0.04
Vlieland (*n* = 64)	Cells	15.17	0.06	− 16.21	0.23	48.69	0.14	14.96	0.06	3.26	0.01
	Plasma	16.59	0.14	− 16.91	0.30	42.03	0.26	9.86	0.09	4.27	0.03
Terschelling (*n* = 60)	Cells	15.01	0.09	− 19.15	0.41	48.72	0.31	15.02	0.10	3.24	0.01
	Plasma	16.56	0.12	− 19.10	0.43	42.72	0.17	9.79	0.06	4.37	0.03
Ameland (*n* = 45)	Cells	15.31	0.17	− 19.07	0.45	49.26	0.28	15.13	0.07	3.26	0.01
	Plasma	16.75	0.19	− 19.25	0.41	42.17	0.16	9.92	0.06	4.26	0.03
Schiermonnikoog (*n* = 83)	Cells	15.52	0.10	− 19.83	0.41	49.32	0.14	15.22	0.05	3.24	0.00
	Plasma	17.21	0.10	− 20.37	0.43	43.33	0.20	10.10	0.05	4.29	0.02

*TN* Total nitrogen (%), *TOC* Total organic carbon (%)

To reconstruct diet composition with stable isotopes, we measured the C and N (δ^13^C and δ^15^N) of blood plasma and RBC of spoonbill nestlings and of the relevant muscle tissue of prey species. All samples were freeze-dried before grinding them with a pestle and mortar. Next, 0.4–0.8 mg of sample material was weighed on a microbalance (Sartorius CP2P) and put into 5 × 8-mm tin capsules. The δ^13^C and δ^15^N isotope values were determined by a Thermo Flash 2000 elemental analyser coupled to a Thermo Delta V isotope ratio mass spectrometer. Isotope values were calibrated to a laboratory acetanilide standard (δ^13^C 26.1‰ and δ^15^N 1.3‰ calibrated to NBS-22 and IAEA-N1, respectively) and corrected for the blank. The results are reported on a per mill scale with respect to Vienna Pee Dee belemnite for δ^13^C and to atmospheric N_2_ for δ^15^N. The replicate error of the standard, acetalinide, ranged between 0.01 and 0.05, when using one standard every 2.2–6.3 samples. The mean diets of all birds were calculated per island for the 2 years combined.

The relative contribution of potential prey species to the diet of spoonbill chicks was estimated using an isotopic Bayesian mixing model programmed in the R-package SIAR version 4.2 (Parnell et al. 2010). The SIAR model requires input of at least two stable isotopes (here δ^15^N and δ^13^C) of a consumer, its prey, and a diet-tissue differentiation factor. As prey sources we used all prey species that occurred at > 2% in the spoonbill diet assessed by regurgitate analysis (Table 1). In order to keep the number of food sources for SIAR low (Phillips et al. 2014), prey that occurred in freshwater or in multiple water types were grouped, since (late-breeding) spoonbills mainly forage on marine Wadden Sea sources (El-Hacen et al. 2014). We did not measure differentiation factors ourselves so we used general ones for avian plasma (δ^15^N, 2.82 ± 0.14‰; δ^13^C, − 0.08 ± 0.38‰) and avian RBC (δ^15^N, 2.25 ± 0.20‰; δ^13^C, − 0.35 ± 0‰) as presented by Caut et al. (2009).

Regurgitates (*n* = 128) produced during the catching and ringing sessions (Fig. 1) were collected individually in separate plastic bags. Regurgitates were stored in a freezer (− 20 °C) on the same day. Single regurgitates were put on a plate for inspection and, with water added, light-weight items such as shrimp tails, uropods, heads, claws, and other whole or almost intact individuals collected first. The remaining light-weight debris was removed by placing the regurgitate in a 800-ml glass beaker filled with water to 600 ml and mixing it with a magnet and magnetic stirrer until all matter was in suspension. To remove the uninformative debris, the mixture in the beaker was carefully overflown by placing the beaker under a slowly running tap. The remaining sample was put on a glass petri dish in order to extract all identifiable parts under a binocular microscope.

The items included otoliths, vertebrae, ventral and dorsal spine, cleithrums, urohyals, bullae, premaxillae, pharyngeal, dentaries, some other bones, insect fragments, crustacean fragments such as heads, carapaces, tails, telsons, uropods, claws (fragments, e.g. dactylus, propodus), legs, swimming pads and skin of amphibian. All parts were classified to the lowest taxonomic level possible, and the size of the parts was used to estimate the length and mass of the individuals (Leopold et al. 2001; C. J. C. et al., unpublished data). Note that, to calculate length and mass from the size of the parts, we used some regression curves developed using larger fish (Leopold et al. 2001), which possibly distorted the estimated length of the small fish. Then, we determined the number of individuals per species, accounting for size and number and orientation of parts per individual.

This study is based on samples collected in the summers of 2012 and 2013. As the sampling of different components (regurgitates, stable isotope values of prey and spoonbills) was not complete in either year, we can not compare the years and present composite values. Unless stated otherwise, the mean and accuracy is given by mean ± SE. Differences in diet between colonies was statistically analysed with ANOVA using Statistica 10, while graphs were made using Sigmaplot 12.3.

## Results

**Fig. 3:**
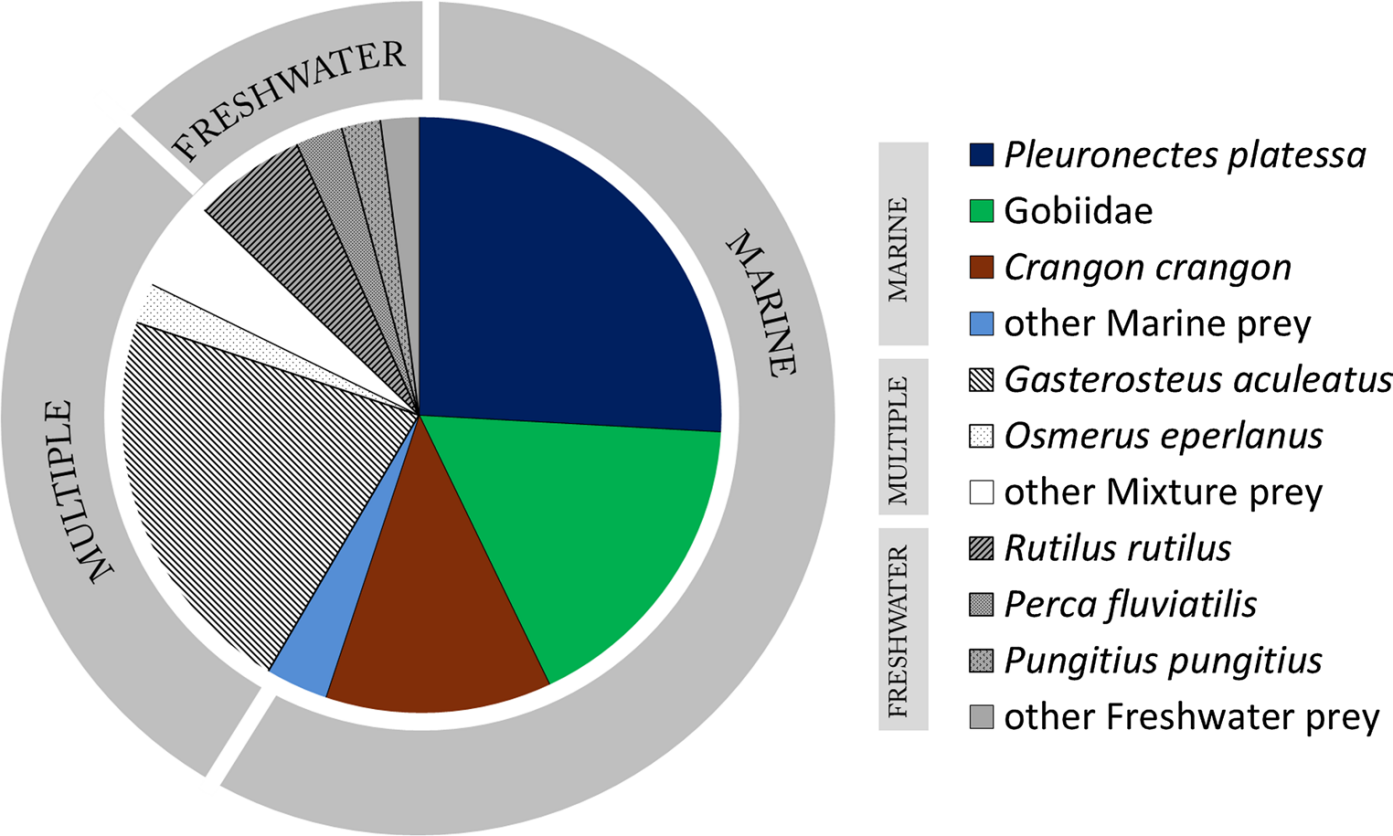
Overall composition in terms of biomass of the diet of nestling Eurasian Spoonbills in the Dutch Wadden Sea based on analysis of regurgitates. Prey are divided into three water type classes: marine prey from the Wadden Sea (marine), prey that occur in more than one water type (multiple), and freshwater prey from waters from the islands or the mainland (freshwater)

The analysis of regurgitates demonstrated that nestling spoonbills on the Wadden Sea islands are fed a great variety of prey of marine and freshwater origin (Table 3). Summarising the information in overall mass terms (Fig. 3), the diet of nestling spoonbills consisted for the greater part (59%) of marine prey from the Wadden Sea. Contrary to expectation, brown shrimp contributed only 12% to this. The main prey species were flatfish (seemingly predominantly plaice) at 26%, three-spined stickleback (22%), gobies (17%). These species had a higher biomass and length, relative to brown shrimp [*Pleuronectes platessa*, biomass 1.07 ± 0.04 g, total length 36.1 ± 0.5 mm (*n* = 1124); *Gasterosteus aculeatus*, biomass 1.40 ± 0.07 g, total length 49.9 ± 0.5 mm (*n* = 637); Gobidae, biomass 0.81 ± 0.04 g, total length 39.9 ± 0.4 mm (*n* = 961); *C. crangon*, biomass 0.19 ± 0.004 1 g, total length (head–tail) 24.3 ± 0.1 cm (*n* = 2391)].

**Table 3:** Spoonbill diet on the barrier islands of the Dutch Wadden Sea in 2012–2013, based on regurgitate analyses

Year: 2012–2013		Texel (*n* = 18)		Vlieland (*n* = 60)		Terschelling (*n* = 15)		Ameland (*n* = 2)		Schiermonnikoog (*n* = 33)		Totals (*n* = 128)	
Habitat	Prey item	Mean	SE	Mean	SE	Mean	SE	Mean	SE	Mean	SE	Mean	SE
Marine	Total	34.2	7.5	55.8	4.3	71.7	6.9	67.7	19.3	69.9	5.3	58.5	3.0
	*Pleuronectes platessa*	20.6	7.1	24.4	3.2	33.5	6.5	25.6	1.2	28.0	3.9	25.9	2.2
	Gobiidae	5.8	1.5	20.6	2.6	17.7	3.3	12.9	4.5	16.2	3.1	16.9	1.6
	Gobiidae	5.8	1.5	20.6	2.6	17.5	3.2	12.9	5.5	16.2	3.1	16.9	1.6
	*Pomatoschistus minutus*	0.0	0.0	0.0	0.0	0.2	0.2	0.0	0.0	0.0	0.0	0.0	0.0
	*Crangon crangon*	7.1	1.2	7.9	1.1	15.5	4.5	7.1	4.0	21.9	2.1	12.3	1.1
	Other marine prey	0.7	0.3	2.9	1.2	5.0	4.1	22.0	17.9	3.9	0.7	3.4	0.8
	*Arnoglossus laterna*	0.0	0.0	0.1	0.1	0.0	0.0	0.0	0.0	0.0	0.0	0.0	0.0
	Carcinidae	0.6	0.3	0.1	0.1	0.0	0.0	1.9	0.0	0.5	0.5	0.3	0.1
	*Carcinus maenas*	0.0	0.0	0.4	0.2	0.6	0.4	0.0	0.0	2.0	0.5	0.8	0.2
	*Cerastoderma edule*	0.0	0.0	0.0	0.0	0.0	0.0	0.0	0.0	0.0	0.0	0.0	0.0
	*Hydrobia ulvae*	0.0	0.0	0.0	0.0	0.0	0.0	0.0	0.0	0.0	0.0	0.0	0.0
	*Liocarcinus holsatus*	0.0	0.0	0.0	0.0	0.1	0.1	0.0	0.0	0.1	0.1	0.1	0.0
	*Littorina littorina*	0.0	0.0	0.1	0.0	0.0	0.0	0.0	0.0	0.0	0.0	0.1	0.0
	*Macoma balthica*	0.0	0.0	0.0	0.0	0.1	0.1	0.0	0.0	0.1	0.0	0.1	0.0
	*Myoxocephalus scorpius*	0.0	0.0	0.1	0.1	4.1	4.1	0.0	0.0	0.0	0.0	0.5	0.5
	*Mytilus edulis*	0.0	0.0	0.0	0.0	0.0	0.0	0.0	0.0	0.1	0.0	0.0	0.0
	*Nereis virens*	0.0	0.0	0.0	0.0	0.0	0.0	0.0	0.0	0.2	0.2	0.1	0.0
	*Pholis gunnulus*	0.0	0.0	1.1	1.1	0.0	0.0	0.0	0.0	0.0	0.0	0.5	0.5
	*Sprattus sprattus*	0.1	0.1	0.8	0.4	0.0	0.0	20.1	0.0	0.9	0.3	0.9	0.4
Mixture^a^	Total	61.5	8.1	32.4	3.7	9.6	3.6	32.3	19.3	12.6	3.6	28.7	2.7
	*Gasterosteus aculeatus*	61.4	8.1	23.3	3.0	8.0	3.3	0.0	0.0	4.2	1.3	21.6	2.5
	*Osmerus eperlanus*	0.0	0.0	2.9	1.3	0.0	0.0	0.0	0.0	3.3	1.6	2.2	0.7
	Other ‘mixture’ prey	0.1	0.1	6.1	2.4	1.6	1.2	32.3	19.3	5.1	2.7	4.9	1.4
	*Anguilla anguilla*	0.0	0.0	0.0	0.0	0.0	0.0	0.0	0.0	4.6	2.7	1.2	0.7
	*Atherina presbyter*	0.0	0.0	0.2	0.1	0.0	0.0	0.0	0.0	0.4	0.4	0.2	0.1
	*Palaemon* sp.	0.1	0.1	2.4	1.6	1.6	1.2	32.3	19.3	0.0	0.0	1.8	0.9
	*Platychtys flesus*	0.0	0.0	1.6	1.6	0.0	0.0	0.0	0.0	0.0	0.0	0.7	0.7
	*Zoarces viviparous*	0.0	0.0	1.9	1.1	0.0	0.0	0.0	0.0	0.0	0.0	0.9	0.5
Freshwater	Total	4.2	4.2	11.8	3.3	18.7	7.3	0.0	0.0	17.5	4.5	12.8	2.2
	*Perca fluviatilis*	0.0	0.0	1.9	0.9	0.9	0.5	0.0	0.0	6.1	2.1	2.6	0.7
	*Pungitius pungitius*	0.0	0.0	4.4	1.9	0.5	0.5	0.0	0.0	0.0	0.0	2.1	0.9
	*Rutilus rutilus*	4.2	4.2	3.8	1.4	14.7	7.2	0.0	0.0	7.9	3.0	6.1	1.5
	Other freshwater prey	0.1	0.0	1.8	1.1	2.6	2.0	0.0	0.0	3.5	1.6	2.1	0.7
	*Abramis brama*	0.0	0.0	0.0	0.0	0.0	0.0	0.0	0.0	0.0	0.0	0.0	0.0
	*Acilius sulcatus*	0.0	0.0	0.0	0.0	0.0	0.0	0.0	0.0	0.0	0.0	0.0	0.0
	*Blicca bjoerkna*	0.0	0.0	0.0	0.0	0.0	0.0	0.0	0.0	0.0	0.0	0.0	0.0
	Callicorixa sp.	0.0	0.0	0.0	0.0	0.0	0.0	0.0	0.0	0.0	0.0	0.0	0.0
	Coleoptera	0.0	0.0	0.1	0.0	0.1	0.0	0.0	0.0	0.0	0.0	0.0	0.0
	Copepoda	0.0	0.0	0.0	0.0	0.0	0.0	0.0	0.0	0.0	0.0	0.0	0.0
	*Corixa punctate*	0.0	0.0	0.0	0.0	0.0	0.0	0.0	0.0	0.0	0.0	0.0	0.0
	Corixa sp.	0.0	0.0	0.0	0.0	0.0	0.0	0.0	0.0	0.0	0.0	0.0	0.0
	*Dytiscus marginalis*	0.0	0.0	0.0	0.0	0.0	0.0	0.0	0.0	0.0	0.0	0.0	0.0
	*Esox lucius*	0.0	0.0	0.0	0.0	0.0	0.0	0.0	0.0	0.0	0.0	0.0	0.0
	*Gobio gobio*	0.0	0.0	0.5	0.5	0.0	0.0	0.0	0.0	0.0	0.0	0.2	0.2
	*Graphoderus* sp.	0.0	0.0	0.0	0.0	0.0	0.0	0.0	0.0	0.0	0.0	0.0	0.0
	*Gymnocephalus cernuus*	0.0	0.0	1.1	0.9	2.5	2.0	0.0	0.0	1.9	0.7	1.3	0.5
	*Notonecta glauca*	0.0	0.0	0.0	0.0	0.0	0.0	0.0	0.0	0.0	0.0	0.0	0.0
	*Orconectes limosus*	0.0	0.0	0.0	0.0	0.0	0.0	0.0	0.0	1.6	1.1	0.4	0.3
	*Sander lucioperca*	0.0	0.0	0.0	0.0	0.0	0.0	0.0	0.0	0.0	0.0	0.0	0.0

Dietary content is expressed as a percentage of the biomass contribution per prey. Prey that occurred at < 2% in the mean Wadden Sea diet (*Totals*) were grouped

^a^Prey that occur in marine, brackish and freshwater habitat types

**Fig. 4:**
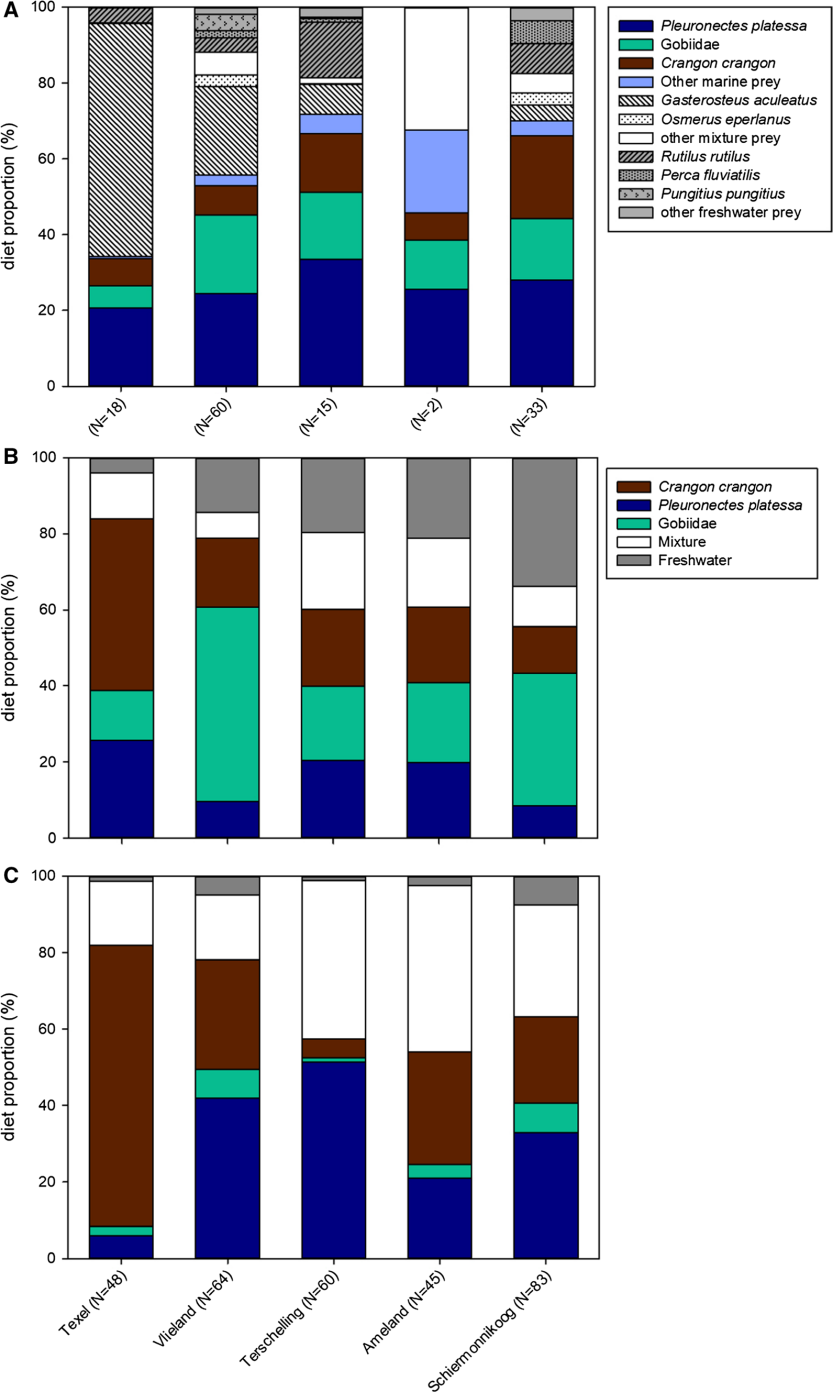
Diet of nestling spoonbills on the different Wadden Sea islands based on **a** regurgitate analysis, **b** stable isotope analysis of plasma, and **c** stable isotope analysis of red blood cells

Apart from the marine prey, the remaining part of the diet consisted of freshwater prey (29%, comprising mostly three-spined sticklebacks; Fig. 3) and prey that could have originated from more than one water type (13%). Figure 4 represents the diet of spoonbill nestlings (regurgitate analysis; Fig. 4a), during the previous few days (isotope analysis based on plasma tissue; Fig. 4b), and that over about a month of nestling life (isotope analysis based on RBC tissue; Fig. 4c) (Rodnan et al. 1957). Restricting the number of sources in SIAR to three (marine, mixture and freshwater) instead of five (Fig. 4b, c), made no meaningful difference to the contributions of marine prey to the diet (mean difference 3.01 ± 2.63%). During the whole nestling period, spoonbill nestlings are mainly fed with marine prey, except for chicks on Texel which mainly had been fed sticklebacks on the day of capture (Fig. 4a) after having been fed a lot of shrimp in the previous weeks (Fig. 4c). Whereas the contribution of flatfish in the diet did not differ between colonies, the contribution of three-spined stickleback decreased from west to east [Fig. 4a, ANOVA _Flatfish_; *F*_(4, 123)_ = 0.668, *p* = 0.615, ANOVA _Stickleback_; *F*_(4, 123)_ = 23.34, *p* < 0.001]. The contribution of gobies varied significantly between islands [ANOVA _Gobiidae_; *F*_(4, 123)_ = 2.51, *p* = 0.045; Fig. 4a] although without the data for Texel, the contribution of gobies was uniform (ANOVA _Gobiidae without Texel_; *F*_(3, 106)_ = 0.489, *p* = 0.690). The isotope-based diet reconstructions confirmed that nestling spoonbills were mainly fed marine prey, with fish (mainly gobies and flatfish) and brown shrimp contributing most to the diet (Fig. 4b, c).

## Discussion

As expected, most prey delivered to growing spoonbill chicks on the barrier islands had a marine origin, indicating that they were caught in the Wadden Sea by the provisioning parents. This means that the growth of chicks is ‘fuelled’ by local prey resources, rather than resources from afar (e.g. found in freshwater habitats on the mainland). Herring gulls *Larus argentatus* breeding in the same areas have been shown to sometimes provision chicks with freshwater food item collected far away in inland areas (Bukacinska et al. 1996). According to a study by El-Hacen et al. (2014), who reconstructed their diet based on feather isotopes, freshwater prey are the main food source for spoonbill chicks on Schiermonnikoog early in the breeding season, and are replaced by marine items later on, matching the time of the year that this study was carried out. For chicks born in June–July 2010, El-Hacen et al. (2014) found a contribution of brown shrimp of 37%, which is more than the SIAR estimates of 23% based on the isotope signature of RBC in the present study (Fig. 4c; Schiermonnikoog).

The finding that flatfish and gobies were the main marine prey species in the Wadden Sea was an unexpected result. After all, the available diet assessments of spoonbills in the Wadden Sea, based on what was taken as common knowledge (Wintermans and Wymenga 1996; Altenburg and Wymenga 1997; Hollander 1997), visual observations of ingested food items (van Wetten and Wintermans 1986a, b), visual examination of the stomach content of a single dead spoonbill (Tinbergen 1933), or direct observations of prey found in feeding areas (Kemper 1986a, b; van Wetten and Wintermans 1986a, b), all indicate that brown shrimp should be the main prey. Indeed, the colour and structure of regurgitates beguilingly suggest brown shrimp to be the main component; this is due to the low digestibility of the shrimps’ chitin exoskeletons (Jackson et al. 1992) compared with the fish meat which is more rapidly digested by the spoonbills.

Our analysis rectifies the notion that shrimp is the main marine prey (at least for the chicks), and suggests that small fish rather than brown shrimp contribute most to the spoonbill nestling diets. Our finding is consistent with prey-preference experiments with a captive second-year spoonbill reported in the grey literature by van Wetten and Wintermans (1986a). When simultaneously offered fish and shrimp, spoonbill preferred fish (van Wetten and Wintermans 1986a). This may be explained by their higher digestibility (Jackson et al. 1992), higher biomass per prey item, and possibly smaller handling times (van Gils et al. 2005). Also, unlike marine fish, shrimp are isotonic with sea water (Spaargaren 1971), yielding a salt load that spoonbills may try to avoid (Gutiérrez 2014; Gutiérrez and Piersma 2016).

From the late 1980s onwards, the Wadden Sea lost a substantial part of its important function as a nursery for flatfish (van der Veer et al. 2011), with small populations of the young age classes of plaice lingering on. Long-term trends in the western Wadden Sea intertidal area are consistent with this view, with a decrease of juvenile flatfish abundance, but without clear trends for gobies and brown shrimp (Jung et al., in review). Furthermore the stocks of adult shrimp in the deeper parts of the Wadden Sea first generally increased (Tulp et al. 2012), followed by a decrease again due to overfishing (Tulp et al. 2016). In view of their preference to provision their chicks with fish rather than shrimp, we suggest that their preferred prey (flatfish) being scarce in recent years will have been the most important factor leading to density-dependent recruitment declines of growing spoonbill colonies and the rapid saturation of colony sizes in the Wadden Sea (Oudman et al. 2017). During the initial phase of their population recovery [1965–1990 (Lok et al. 2013)], spoonbills might actually have benefited from the favourable food conditions in the form of an abundance of juvenile flatfish and gobies rather than mature brown shrimp (van der Veer et al. 2011), compared to relatively greater densities of brown shrimp recently.

The current levelling off of the growth of the spoonbill population breeding on the Wadden Sea barrier islands (Oudman et al. 2017) is associated with low stocks of their favourite small fish prey (van der Veer et al. 2011). A preference for small fish rather than shrimp would make colony growth characteristics good indicators of the abundance of small fish in the Wadden Sea. This is a state of affairs that appears comparable to that of the harbour seals (*Phoca vitulina*) in the Dutch Wadden Sea, where population levelling off is also explained by limited access to (larger) fish (Brasseur et al. 2018). We predict that successful (fishery) management towards recovery of the former densities of young flatfish, or an increase of small and juvenile fish abundance in general, will be tracked by changing spoonbill diets (more fish), improved breeding success, and an increase in the size of spoonbill colonies across the Wadden Sea.
